# Autologous olfactory mucosa mesenchymal stem cells treatment improves the neural network in chronic refractory epilepsy

**DOI:** 10.1186/s13287-023-03458-6

**Published:** 2023-09-07

**Authors:** Zheng-Zhao Liu, Yan Huang, Chun-Gu Hong, Xin Wang, Ran Duan, Jian-Yang Liu, Jia-Lin He, Da Duan, Hui Xie, Ming Lu

**Affiliations:** 1 Department of Neurosurgery, the 921st Hospital of PLA (Second Affiliated Hospital of Hunan Normal University), Changsha, 410081 Hunan China; 2 Department of Orthopedics, Xiangya Hospital, Central South University, Changsha, 410008 Hunan China; 3 Movement System Injury and Repair Research Center, Xiangya Hospital, Central South University, Changsha, 410008 Hunan China; 4 Department of Sports Medicine, Xiangya Hospital, Central South University, Changsha, 410008 Hunan China; 5 Hunan Key Laboratory of Organ Injury, Aging and Regenerative Medicine, Changsha, 410008 Hunan China; 6 Hunan Key Laboratory of Bone Joint Degeneration and Injury, Changsha, 410008 Hunan China; 7 National Clinical Research Center for Geriatric Disorders, Xiangya Hospital, Central South University, Changsha, 410008 Hunan China; 8 NHC Key Laboratory of Birth Defect for Research and Prevention, Hunan Provincial Maternal and Child Health Care Hospital, Changsha, 410008 Hunan China; 9 Hunan Provincial Key Laboratory of Neurorestoration, Hunan Normal University, Changsha, 410081 Hunan China; 10 Key Laboratory of Protein Chemistry and Developmental Biology of Ministry of Education, College of Life Sciences, Hunan Normal University, Changsha, 410219 Hunan China; 11 First Clinical Department of Changsha Medical University, Changsha, 410081 Hunan China; 12 Department of Neurology, The Second Xiangya Hospital, Central South University, Changsha, 410011 Hunan China

**Keywords:** Autologous transplant, Olfactory mucosa mesenchymal stem cell, Refractory epilepsy, Diffusion cerebral atrophy, Treg cells, Inflammation, Neural network

## Abstract

**Background and aims:**

Refractory epilepsy is also known as drug-resistant epilepsy with limited clinical treatment. Benefitting from its safety and easy availability, olfactory mucosa mesenchymal stem cells (OM-MSCs) are considered a preferable MSC source for clinical application. This study aims to investigate whether OM-MSCs are a promising alternative source for treating refractory epilepsy clinically and uncover the mechanism by OM-MSCs administration on an epileptic mouse model.

**Methods:**

OM-MSCs were isolated from turbinal and characterized by flow cytometry. Autologous human OM-MSCs treatment on a patient was carried out using intrathecal administration. Epileptic mouse model was established by 1 mg/kg scopolamine and 300 mg/kg pilocarpine treatment (intraperitoneal). Stereotaxic microinjection was employed to deliver the mouse OM-MSCs. Mouse electroencephalograph recording was used to investigate the seizures. Brain structure was evaluated by magnetic resonance imaging (MRI). Immunohistochemical and immunofluorescent staining of GFAP, IBA1, MAP2, TUBB3, OLIG2, CD4, CD25, and FOXP3 was carried out to investigate the neural cells and Treg cells. QRT-PCR and ELISA were performed to determine the cytokines (*Il1b, Il6, Tnf, Il10*) on mRNA and protein level. Y-maze, the object location test, and novel object recognition test were performed to measure the cognitive function. Footprint test, rotarod test, balance beam test, and grip strength test were conducted to evaluate the locomotive function. Von Frey testing was carried out to assess the mechanical allodynia.

**Results:**

Many beneficial effects of the OM-MSC treatment on disease status, including seizure type, frequency, severity, duration, and cognitive function, and no apparent adverse effects were observed at the 8-year follow-up case. Brain MRI indicated that autologous OM-MSC treatment alleviated brain atrophy in epilepsy patients. A study in an epileptic mouse model revealed that OM-MSC treatment recruited Treg cells to the brain, inhibited inflammation, rebuilt the neural network, and improved the cognitive, locomotive, and perceptive functions of epileptic mice.

**Conclusions:**

Autologous OM-MSC treatment is efficacious for improving chronic refractory epilepsy, suggesting a future therapeutic candidate for epilepsy.

*Trial registration*: The study was registered with Chinese Clinical Trial Registry (ChiCTR2200055357).

**Supplementary Information:**

The online version contains supplementary material available at 10.1186/s13287-023-03458-6.

## Background

Although several anti-epileptic drugs (AEDs) are effective for epilepsy treatment, almost 30% of epileptic patients still fail to achieve good control with AED intervention [1]. At present, dietary, immunological, surgical treatments, and deep brain stimulation appear to be beneficial for treating AED-resistant patients. However, many patients are still not suitable for these approaches [2, 3]. Studies of animal models of epilepsy have demonstrated that neural cell and gene therapies have a beneficial effect on epilepsy [4, 5]. However, delayed gene expression after transfection and differentiation of neural cells reduced the efficacy of this approach for acute seizures [6]. New ways to treat this refractory epilepsy are therefore needed.

Benefitting from safety and clinical efficacy, growing concern has arisen about MSCs in epilepsy treatment. However, source limitations and invasive procedures restrict the application of MSCs from bone marrow, umbilical cord, adipose tissue, and dental [7–9]. Lu and coworkers recently isolated MSCs from the nasal olfactory mucosa (OM) [10]. Compared with other MSCs, OM-MSCs have many attributes and are considered to be a preferred cell type for epilepsy treatment. First, OM-MSCs can be obtained from a convenient, safe, and cost-effective procedure [11]. It is one of the few places in the nervous system accessible under local anesthetic and noninvasive endoscopic surgical procedures [12]. Second, OM-MSCs display multilineage differentiation, permanent proliferation, and easy culture in vitro [13, 14]. Furthermore, OM-MSCs are cultured with the patient's own serum and transplanted in an autologous way, and immunological rejection could be avoided and not susceptible to tumorigenicity or chromosomal abnormality either in vivo or in vitro [15]. Thus, there are no ethical concerns regarding the use of OM-MSCs. Unlike other mesoderm-derived MSCs, OM-MSCs originate from ectoderm and are favorable for neural differentiation. OM-MSCs show great potential to rebuild the neural network by modulating the immunosystem [16]. These features make OM-MSCs a promising cell source for the treatment of neurological disorders.

Here, we first demonstrate that OM-MSCs have beneficial effects for epilepsy treatment, evidenced both from a clinical treatment of chronic refractory epilepsy and an epilepsy mouse model, showing improved conditions of epilepsy following OM-MSC treatment. The beneficial effects on the neural network were also demonstrated.

## Materials and methods

### OM-MSCs preparation and transplantation in human

Autologous OM-MSCs were prepared as previously published protocol [11, 15]. According to the regulations of Chinese Pharmacopoeia, the testing procedures for bacteria, viruses, fungi, mycoplasma, and endotoxin were followed. Before transplant, the isolated OM-MSCs (total volume of 2 mL) (5 × 10^7^ cells/ml) were prepared, which were characterized by the CD44-, CD105-, CD133-, CD146-, CD73-, and CD90-positive and CD45- and CD34-negative. After culture expansion in vitro, OM-MSCs were transplanted to the patient through lumbar puncture using 1 × 10^6^ cells/kg. The patient received autologous OM-MSCs transplant twice at 1-week interval on April 22, 2014, and April 28, 2014. The patient did not show any severe adverse reactions or complications to the intrathecal administrations of OM-MSCs.

### Flow cytometry

Mouse OM-MSCs were obtained from turbinal of wild-type or GFP transgenic mice as previously described [17]. After culture expansion in vitro, the isolated OM-MSCs were stained with anti-CD45 (BD, 553079), anti-CD34 (BD, 560238), FITC-conjugated anti-CD31 (R&D, FAB3628G), PE-conjugated anti-CD73 (BD, 550741), anti-CD105 (BD, 562761), and APC-conjugated anti-CD90 (R&D, FAB7335R). Isotype control was used to determine cell gating. One hundred thousand cells/sample were collected using FACS Canto II (BD). We analyzed data using FlowJo V10.5.3 software.

### Chronic epileptic mouse model

Male mice (C57BL/6, weighing 18–25 g, 8 weeks old) were obtained from the Hunan SJA Laboratory animal Co., Ltd. There were four groups of mice randomly assigned (*n* = 10 per group): Vehicle group, Vehicle + OM-MSCs group, Pilocarpine (Pilo) group, and Pilo + OM-MSCs group. At 22 °C and 50% humidity, mice were housed in gnotobiotic isolators with a 12-h light–dark cycle. The pilocarpine epilepsy mouse model was established. Briefly, mouse was pretreated with 1 mg/kg scopolamine (intraperitoneal injection) for 30 min, followed by 300 mg/kg pilocarpine administration (intraperitoneal injection). When the modeling was successful, mouse’s limbs were convulsed within 30 min, and seizures occurred in 2 h. The status epilepsy (SE) lasted for 24 h, followed by the latent period without spasms (4–44 days) and then the chronic period with 2–3 spasms a week. We administrated the OM-MSCs transplantation (brain stereotaxic injection) during the latent period.

### Brain stereotaxic injection

Brain stereotaxic microinjection was performed using Yuyan stereotaxic instrument (Shanghai, China) as previously described [18]. Briefly, after anesthetizing the mice with 0.3% pentobarbital sodium (20 µL/g), mice were placed on the platform. Followed by shaving mice’s hair on skull and incising the skin along the midline, the hippocampal location was marked (1.8 mm beside the sagittal suture, 2.3 mm below the bregma, 2.0 mm in depth). The periosteum was digested with 3.0% H_2_O_2_, and the hole was drilled with a 0.5-mm drill bit. 2 µL 1 × 10^5^ cells/μL OM-MSCs were injected into the hippocampus with a flow rate of 1.0 µL/3 min using a syringe on the positioner. The skin was sutured and disinfected with iodophor.

### Mouse electroencephalograph (EEG) recording

Anode and cathode were implanted in 2.3 mm backward from bregma, 2.0 mm lateral from the midline (left and right separately), and 2 mm in depth from subdural. Another reference electrode was placed over the cerebellum. Briefly, the mouse was anesthetized with 0.3% pentobarbital sodium (20 µL/g). After shaving mice's hair on the skull, we drilled holes in the skull, inserted the screws, and fixed with dental cement. Multichannel Physiological Signal Acquisition and Processing System [RM-6240E] [19, 20] (Chengdu, China) was connected to mice with wires. The implants remained affixed and produced good recordings for over a month. We recorded the EEG 10 min/time and with 15-min interval for three times/day. The statistics on amplitude of EEG is shown with five positive times during seizures in Pilo group. We calculated the average of amplitude within the recorded 10 min *n* = 5 per group.

### Magnetic resonance imaging (MRI)

Raysolution Co., Ltd. (Wuhan, Hubei, China), performed MRI using a 7T Bruker Biospec 70/30 system (Bruker Biospin, Ettlingen, Germany). The mice were anesthetized and sedated using 2% isoflurane equipped with Matrix VIP 3000 (Midmark, Ohio, USA). Respiration and body temperature (37 °C) were monitored. Body temperature was maintained at 37 °C with a water circulation. T2 measurements were taken across 30 contiguous slices using the following parameters: slice thickness = 0.4 mm, repetition time = 4786.93 ms, and echo time = 45 ms. High T2 values mean fluid accumulation in the extracellular space in T2-weighted images (white). And the average of three contiguous slices was used for measuring the ventricle volume analyzed by Image-Pro Plus 6.0. Relative ventricle area was analyzed by calculating percentage of the ventricle area (white) in the whole image.

### Brain procurement

One month after OM-MSC implantation, mice were euthanized. Subjects were deeply anesthetized with 0.3% pentobarbital sodium (20 uL/g) and transcardially perfused with saline (30 mL) and subsequently with 4% paraformaldehyde (40 mL). We quickly removed the brains and fixed them in 4% paraformaldehyde for 4–6 h.

### Immunohistochemistry

IHC-paraffin protocol from Abcam was applied: dehydrating brain tissues in graded ethanol, embedding in paraffin, cutting into 4-μm slices, deparaffinizing, rehydrating, followed by antigen retrieval, immunohistochemical staining, dehydration, mounting medium stabilization, and microscopy viewing. Slices were incubated with anti-OLIG2 (Zsbio, ZA-0561) antibody at 4 °C overnight. We rinsed the sections three times with PBS (5 min for each) and then applied the secondary antibodies at room temperature against rabbit IgG HRP (horseradish peroxidase) (Sigma-Aldrich, AP307P; 1:200) for 2 h, followed by washing with PBS three times. We incubated them with chromogenic substrate DAB (Sigma-Aldrich, D3939) for 2–8 min for chromogenic reaction and then sealed with neutral resin for microscopic detection. Staining intensity times cell percentage was used to calculate staining score: (+++) % × 3 + (++) % × 2 + (+) % × 1 + (–) % × 0, where +++ denotes strong staining signal; ++ denotes medium staining signal; + indicates weak staining signal; and – indicates negative.

### Immunofluorescence

Liquid nitrogen was used to quickly freeze tissues embedded in OCT for frozen section examination. The slices were sectioned using a cryostat microtome (5.0 μm) and incubated overnight at 4 °C with an anti-GFAP antibody (Bioss, bs-0199R). Following three rinses (5 min per time) with 0.1 M PBS, they were incubated with secondary antibodies for 1h , then rinsed three times with PBS, and mounted with antifade mounting medium and DAPI (VECTOR, H-1200). ZEISS ApoTome.2 Imaging System (Germany) was used to obtain the pictures. Immunofluorescent staining of CD25 (eBioscience, MA5-12680), CD4 (eBioscience, 14-9766-82), FOXP3 (CST, 98377S), IBA1 (Abcam, ab178847), TUBB3 (Proteintech, 66375–1-Ig), and MAP2 (Proteintech, 17490-1-AP) in the brain tissue was carried out following the same protocol.

### qRT-PCR

RNA was extracted using the Trizol method, followed by reverse transcription and quantitative real-time PCR [18]. cDNA was synthesized using GoScript™ Reverse Transcriptase (Promega, A5001). Primers were synthesized in Sangon Biotech (Shanghai, China). The QRT-PCR was performed on an FTC-3000 real-time PCR machine using GoTaq® qPCR Master Mix (Promega, A6001). *Gapdh* was served as an internal control. Following denaturation (2 min at 95 °C), 40 cycles of 15 s at 95 °C and 60 s at 60 °C were performed to amplify the product.

**Table:** 

Primer name	Sequence
m-*Gapdh*-F	5′-AGGTCGGTGTGAACGGATTTG-3′
m-*Gapdh*-R	5′-TGTAGACCATGTAGTTGAGGTCA-3′
m-*Tnf*-F	5′-CATCTTCTCAAAATTCGAGTGACAA-3′
m- *Tnf*-R	5′-TGGGAGTAGACAAGGTACAACCC-3′
m-*Il1b*-F	5′-AAGGAGAACCAAGCAACGACAAAA-3′
m-*Il1b*-R	5′-TGGGGAACTCTGCAGACTCAAACT -3′
m-*Il6*-F	5′-GAGGATACCACTCCCAACAGACC-3′
m-*Il6*-R	5′-AAGTGCATCATCGTTGTTCATACA-3′
m- *Il10*-F	5′-ATAACTGCACCCACTTCCCAGTC-3′
m- *Il10*-R	5′-CCCAAGTAACCCTTAAAGTCCTGC-3′

### Enzyme-linked immunosorbent assay (ELISA)

Cytokines in the serum were measured by the Mouse TNF-A Valukine ELISA Kit (VAL609, R&D, USA), Mouse IL-6 Valukine ELISA Kit (VAL604, R&D, USA), and Mouse IL-1β Valukine ELISA Kit (VAL601, R&D, USA). We measured the absorption using the Varioskan LUX multimode microplate reader from Thermo Fisher Scientific (USA). The cytokine concentration in pg/mL sample volume was calculated by fitting a standard curve to each analysis.

### Y-maze and testing procedure

Y-maze was performed to measure the response to novelty. The apparatus is black with three arms each 34 cm long, 8 cm wide, and 15 cm high (start arm [SA], novel arm [NA], other arm [OA]). For habitation phase, the mouse was put into SA and OA for 10 min. 1 h later, for trial phase, the mouse was put into the SA with its head pointing away from the center of the maze and allowed to visit the three arms of the maze for 5 min. During each trial, visits to each arm were recorded and the percentages of visits and time spent in NA arm were calculated [% of visit of novelty = the total number of entries to NA/(SA + NA + OA); % of time spend on novelty = the total time spend on NA/(SA + NA + OA)]. In the trial phase, significant exploration of novelty was defined by a duration and/or number of new arm visits of more than 40%. The number of arm visits during the 5-min trial was used to assess locomotor activity.

### The object location test (OLT) and novel object recognition test (NORT)

OLT and NORT were performed following previously description [21]. This space is constrained within a square polymethyl methacrylate box (40 cm 40 cm × 40 cm) that has different colors on the four walls (external cues) to help mice in resolving this spatial memory. The cues were kept in a constant location during testing. A pair of identical black cylinders (5 cm in diameter, 10 cm tall) was placed 6 cm away from the side wall at two corners of the device. As part of habituation, the mice explored the instrument freely for 2 min without any objects for 3 consecutive days. Two identical subjects were placed in the sample test (T1), and the mice were allowed to explore freely for 2 min. For OLT, one mouse moved one object to a new location (NL), while the other stayed in a familiar place (FL). For NORT, a cube was substituted (NO, 5 cm × 5 cm × 5 cm) for one cylinder, while the other cylinder was left remained (FO). We conducted the selection test (T2) by allowing mice to explore for 2 min and recording the exploring time of FL/FO or NL/NO. Mice’s nose points toward the object were no more than 2 cm defined as exploration. Staying on the object or turning around was not regarded as exploration. We recorded the time spending in exploring each object during T1 and T2. Discrimination index: (NL − FL)/(NL + FL) or (NO − FO)/(NO + FO); % Investigation time of novel object: NO/(NO + FO); % Investigation time of novel location: NL/(NL + FL). Here, T1 means the exploring time in T1 and T2 means the exploring time in T2.

### Footprint test

The mice's gait was tested using footprints as previously described [18]. Using the non-toxic waterproof paint, the forepaws of the mouse were painted red and the hind paws were painted black. In order to record the footprints, mice were placed on one end of the tunnel (10 cm × 10 cm × 70 cm). An additional paper (white) is placed on the bottom for recording the footprints. The overlap between hindlimb and forelimb, hind base width, front base width, hindlimb stride length, and forelimb stride length and speed to cross the tunnel were recorded for analysis.

#### Rotarod

Balance and motor coordination were measured using an accelerating rotarod as reported previously [18]. A rotarod with an accelerating force was used for the experiment (DXP-3, Chinese Academy of Medical Sciences). The rotarod was set to accelerate from 3 to 60 rpm for 2 min and then held at 60 rpm for another 2 min. The latency to fall was measured. During a 5-day period, mice were measured three times per day with an interval of 20 min.

### Grip strength test

Muscle tension was examined by grip strength test as reported previously [18]. 40 cm above the horizontal surface of the ground, a steel wire (9 mm in diameter and 50 mm in length) was placed. Mice were allowed to grip the wire. Over a period of 5 min, latency to fall off the wire was measured. During a 5-day period, each mouse was given three trials each day with a 20-min interval.

### Balance beam test

The ability of the mice to maintain balance was evaluated as previously described [18]. 40 cm above ground, a horizontal wooden bar (0.9 cm × 0.9 cm × 50 cm) was placed above the cage. A dark goal box was placed on the other end of the wooden bar. The speed to cross the beam was calculated based on the time taken to traverse the beam. Mice were assessed three times each day with a 20-min interval, which was repeated for 5 days.

### Von Frey testing

The mechanical allodynia test of the hind limb was performed using von Frey filaments (IITC, Woodland Hills, CA) as reported previously [22]. Mouse was allowed to accommodate for 15 min in plastic cages with a wire mesh bottom. The plantar surface of the hind paw was treated with von Frey filaments (0.04–6.0 g, starting with 0.4 g) perpendicularly until it just bent and then held in place for 3 s. If the mouse shook the paw, licked, or withdrew, a positive response was reported and the von Frey threshold (g) was recorded. Up–down iterative method was used to determine the withdrawal threshold. Tests were conducted blindly, where the investigator was unaware of both animal identification and study groups.

### Statistical analysis

Averaging and standard deviations were calculated using Prism 7 (GraphPad Software). Two groups with one variance were compared using an unpaired *t* test. To check the distribution style, a Shapiro–Wilk normality test was performed. An ANOVA test with one way was conducted for studies involving only one variance, and a two-way ANOVA was conducted for studies involving multiple variances. A post hoc analysis of Dunnett's multiple comparison tests was performed to determine whether there was a statistically significant difference between the two groups. *P* value < 0.05 was statistically significant, and ns means no significance, *P < 0.05, **P < 0.01, ***P < 0.001, ****P < 0.0001.

## Results

### Autologous OM-MSC treatment alleviated brain atrophy and improved seizure symptoms in a chronic refractory epilepsy patient

**Fig. 1:**
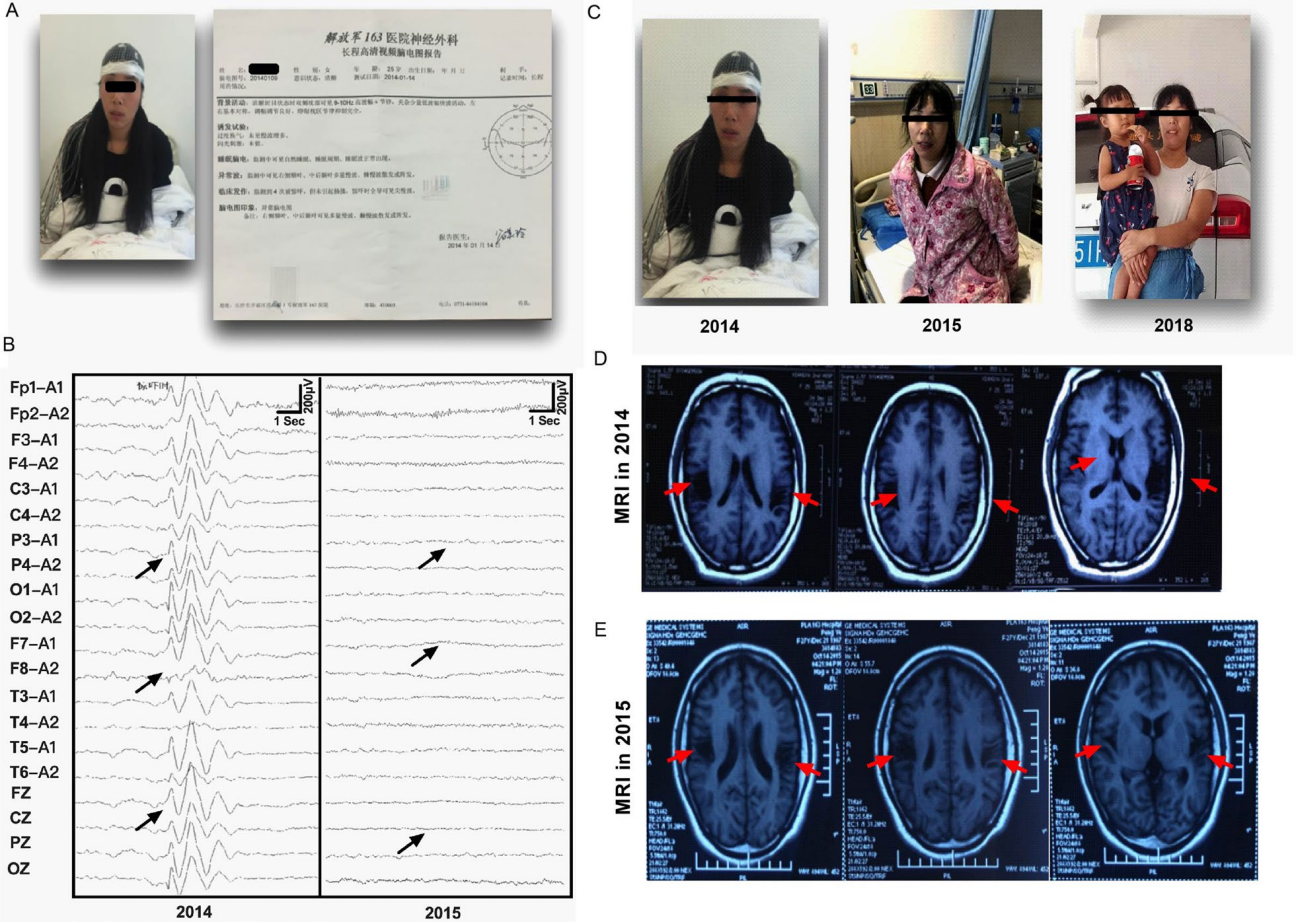
Clinical data of patient before and after autologous OM-MSC therapy. **A** A 26-year-old female suffered from chronic refractory seizures for 17 years. **B** The ambulatory electroencephalography (AEEG) was recorded the process of epileptogenesis in 2014 and 2015, before and after autologous OM-MSC therapy. **C** The clinical condition of patient by taking pictures in 2014 before autologous OM-MSC therapy, and follow-up in 2015 and 2018 after autologous OM-MSC therapy. **D** Brain magnetic resonance imaging (MRI) showed obviously focal malacia localizing in the bilateral temporal region as well as moderate generalized cerebral atrophy in 2012 before autologous OM-MSC therapy. **E** MRI showed no obviously changes in intracranial structure in 2015 relative to in 2014 (before OM-MSCs transplantation)

A 26-year-old married female suffered from chronic refractory seizures for 17 years. She began with generalized tonic–clonic seizures (GTCSs), which were characterized by tonic spasms of limbs and sudden loss of consciousness. Epileptic seizures lasted for 3–5 min each time and occurred 6–8 times per month. However, the seizure frequency of GTCSs was significantly increased to 40 times a month after the birth of her first child in 2012, and the duration of GTCSs was up to 5–7 min per time. Meanwhile, the patient appeared absence seizure and lasted for 25–35 s every time. Moreover, even the slightest noise, such as honking, or emotional stimulation can trigger seizures symptoms. The patient also experienced recurrent headaches and cognitive impairment, which seriously affect the quality of life of the patient (Fig. 1A, Additional file 8: Table S1). Ambulatory electroencephalography (AEEG) was used to record the process of epileptogenesis (Fig. 1B). Brain magnetic resonance imaging (MRI) showed an obvious focal malacia localized in the bilateral temporal region as well as moderate generalized cerebral atrophy. We speculated that cerebral tissue softening in the right temporal lobe may be responsible for epileptic seizures. Thus, clinically, this patient was diagnosed with chronic refractory epilepsy (RE).

Autologous OM-MSCs were isolated from olfactory mucosa and characterized as CD44-, CD105-, CD133-, CD146-, CD7-3-, and CD90-positive and CD45- and CD34-negative (Additional file 1: Fig. S1). After culture expansion in vitro, OM-MSCs were transplanted to the patient through lumbar puncture at 1 × 10^6^ cells/kg. The patient received autologous OM-MSCs transplantation twice at 1-week intervals on April 22, 2014, and April 28, 2014. During and after treatment, we monitored the patient's vital signs, including temperature, respiration, pulse, blood pressure, and electrocardiogram. The patient was afebrile, with stable vital signs. The patient did not show apparent severe adverse reactions or complications to the intrathecal administration of OM-MSCs.

We assessed the changes in the disease status of the patient in the next 8 years (from 2014 to 2021). In this follow-up term, status was recorded in 2014 (before OM-MSC transplantation treatment), 2015 (1 year after OM-MSC transplantation), and 2018 (3 years after OM-MSCs transplantation) (Fig. 1C). Follow-up results showed improved conditions of patient, including physical, intellectual and sleep status. There were no adverse events such as neurological worsening or evidence of tumors after OM-MSC transplant therapy.

**Fig. 2:**
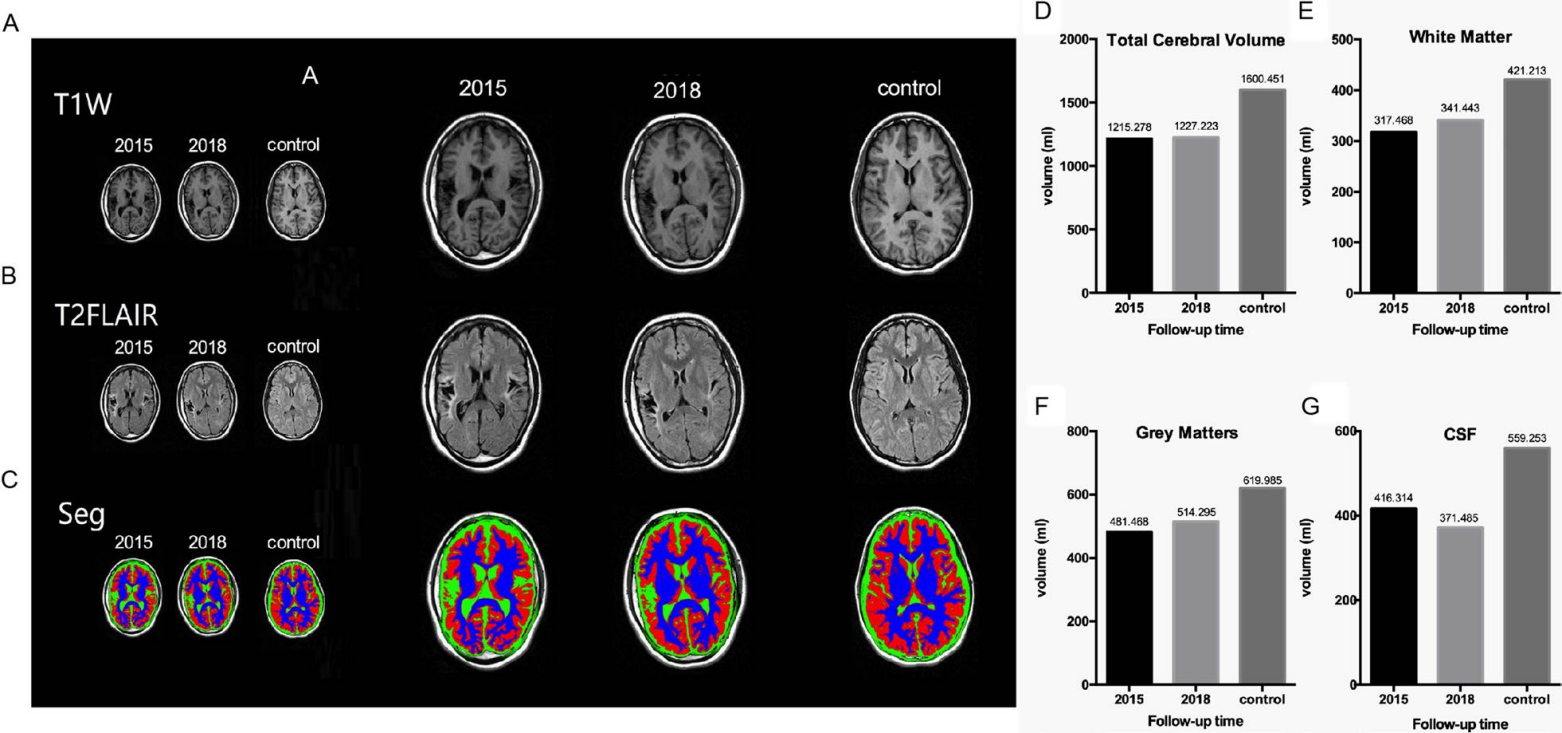
The improvement in cerebral atrophy in follow-up after autologous OM-MSC treatment.** A** T1-weighted cerebral MRI showing in 2015, 2018, and normal control. **B** T2-FLAIR cerebral MRI showing in 2015, 2018, and normal control. **C** The automatic brain tissue segmentation imaging showing in 2015, 2018, and normal control, white matters (blue), gray matters (red), CSF (green). Normal control represented a healthy 26-year-old female cerebral MRI. **D–G** Representative data demonstrating brain anatomy images and cerebral tissue segmentation results obtained from 1-year follow-up in 2015 and 4-year follow-up in 2018. **D** Total cerebral volume (TCV) increased by 0.98% from 2015 to 2018. **E** White matter volume increased by 7.54% from 2015 to 2018. **F** Gray matter volume increased by 6.81% from 2015 to 2018. **G** CFS volume decreased by 10.76% from 2015 to 2018. Normal control represented a healthy 26-year-old female cerebral volume

The patient exhibited transformations of her GTCS to absence seizure (AS). In particular, improvement in the frequency and duration time were observed in 2014 after OM-MSC transplantation. Epilepsy symptoms in patient were significantly improved in at the 1-year follow-up in 2015. The GTCSs were no longer occurred. Meanwhile, the symptoms of absence seizures were obviously improved. The absence seizure frequency was reduced to 2–3 times a day, every time lasting for 10–12 s. Surprisingly, the patient did not need to take any AEDs in 2015 and delivered a second healthy baby. Meanwhile, abnormal brain waves, including spike-sharp waves and slow waves, were significantly reduced after OM-MSC treatment, as evidenced by EEG recording in 2015 (Fig. 1B). Cognitive status was also alleviated (the MMSE score was 24/30). Further, the patient's condition was more stable and still dominated by the absence of attacks, with an average of 10–25 attacks per year, each lasting 6–8 s from 2016 to 2018. There was no obviously improvement in brain MRI in 2015 (Fig. 1D, E). Brain MRI still showed diffuse cerebral atrophy with white and gray matter paucity, enlargement of lateral ventricles and subarachnoid space, and deep gyri and widened sulci at the 1-year follow-up in 2015. However, follow-up cerebral MRI obtained 4 years in 2018 after autologous OM-MSC therapy showed dramatic reversibility of diffusion brain atrophy exhibited in MRI (Fig. 2A, B). To further identify the changes in brain volume after OM-MSC transplantation, we used a well-established automatic brain tissue segmentation method to calculate the brain volume (Fig. 2C, Additional file 2: Fig. S2). The total cerebral volume (TCV) was calculated by combining total gray matter, white matter, and CSF as defined by automatic tissue segmentation. TCV increased by 0.98% from the 1-year to 4-year follow-up. The volume of white and gray matter increased by striking 7.54% and 6.81%, respectively, whereas the content of CSF decreased by 10.7% from 2015 to 2018 (Fig. 2D–G). Therefore, the present data suggested that autologous OM-MSC treatment alleviated brain atrophy and improved seizure symptoms in this chronic refractory epilepsy patient.

### OM-MSCs alleviated epileptic seizures in a chronic epileptic mouse model

**Fig. 3:**
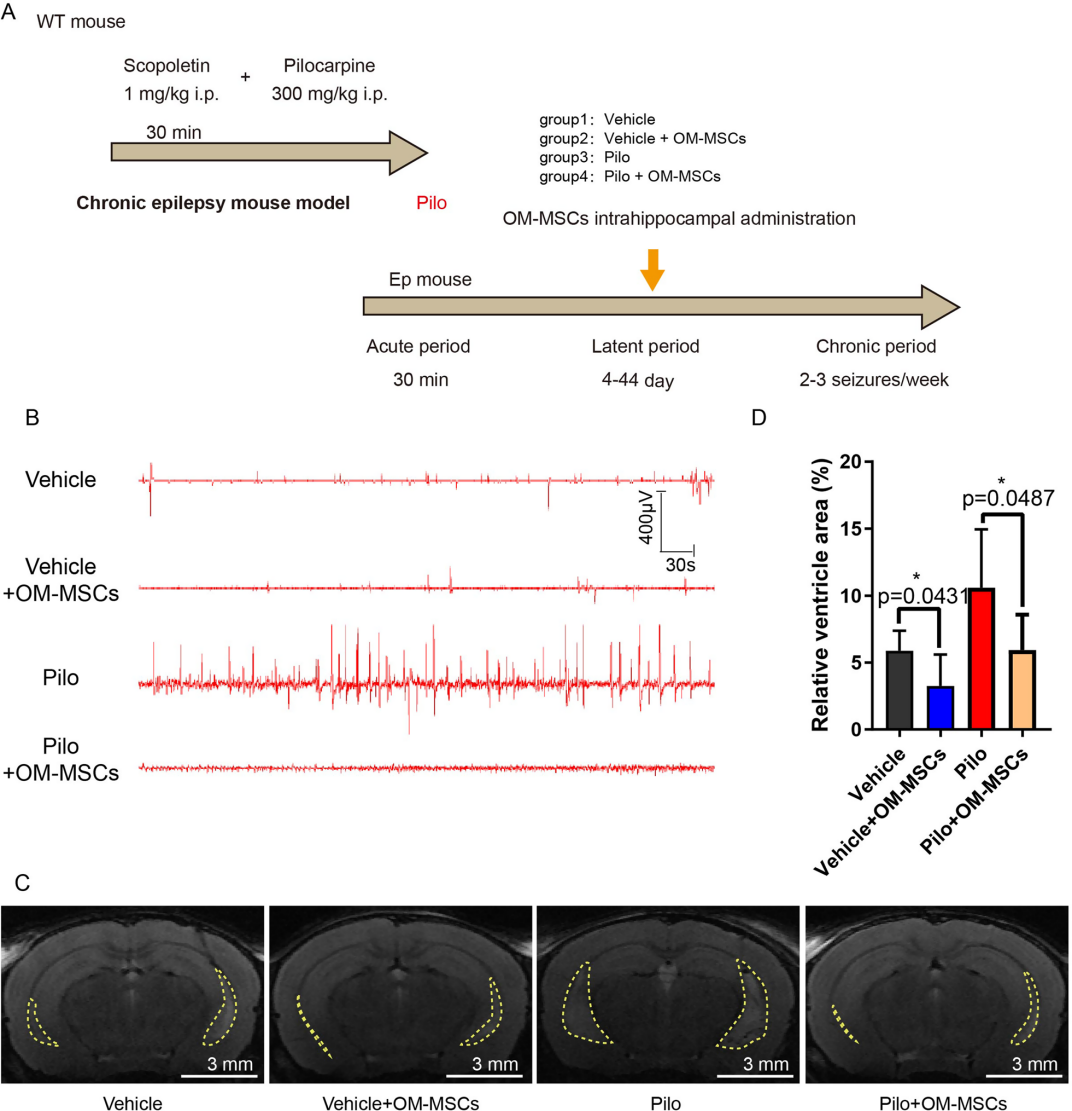
OM-MSC treatment improves cerebral atrophy in epileptic mouse model.** A** Schematic representation of experimental procedures. OM-MSCs were delivered using brain stereotaxic injection during the latent period. **B** Representative EEG demonstrating epileptiform activity in WT (Vehicle), epileptic (Pilo), and OM-MSC treated (Vehicle + OM-MSCs, Pilo + OM-MSCs) mice. *n* = 10 per group. **C** Representative MRI images and lateral ventricles (enclosed by dashed line). Scale bar: 3 mm. **D** Quantitative MRI analysis of relative ventricle area. *n* = 3 per group

To evaluate the beneficial effect of OM-MSCs on epilepsy, mouse OM-MSCs were isolated from turbinal of mouse and characterized as CD45-, CD34-, and CD31-negative and CD105-, CD90-, and CD73-positive (Additional file 3: Fig. S3). We generated a chronic epileptic mouse model and transplanted mouse OM-MSCs into the mouse hippocampus during the latent period by stereotaxic delivery (Fig. 3A). Using OM-MSCs isolated from GFP transgenic mice, OM-MSC-GFP could be observed in the hippocampal region after 7-day and 14-day transplantation (Additional file 4: Fig. S4), which indicated distribution of OM-MSCs in hippocampus through stereotaxic delivery. EEG recordings demonstrated that the amplitude and frequency were markedly lower in the Pilo + OM-MSC group than in the Pilo group (Fig. 3B, Additional file 5: Fig. S5). Upon OM-MSCs treatment, the amplitude of EEG recorded from epileptic mice almost reduced to the comparable level of wild type. To examine the brain structure after OM-MSC transplantation, T2-weighted MRI was employed. MRI images of the brain revealed that enlarged ventricle on epileptic mice was improved after OM-MSC treatment (Fig. 3C, D). Postmortem histology of the brain exhibited the alleviated damage to the brain of epileptic mice after OM-MSC treatment (Additional file 6: Fig. S6). These results recapture the beneficial effects of OM-MSCs to reduce the frequency of seizures and improve the brain atrophy.

### OM-MSCs inhibit inflammation and restore the neuronal cell lose

**Fig. 4:**
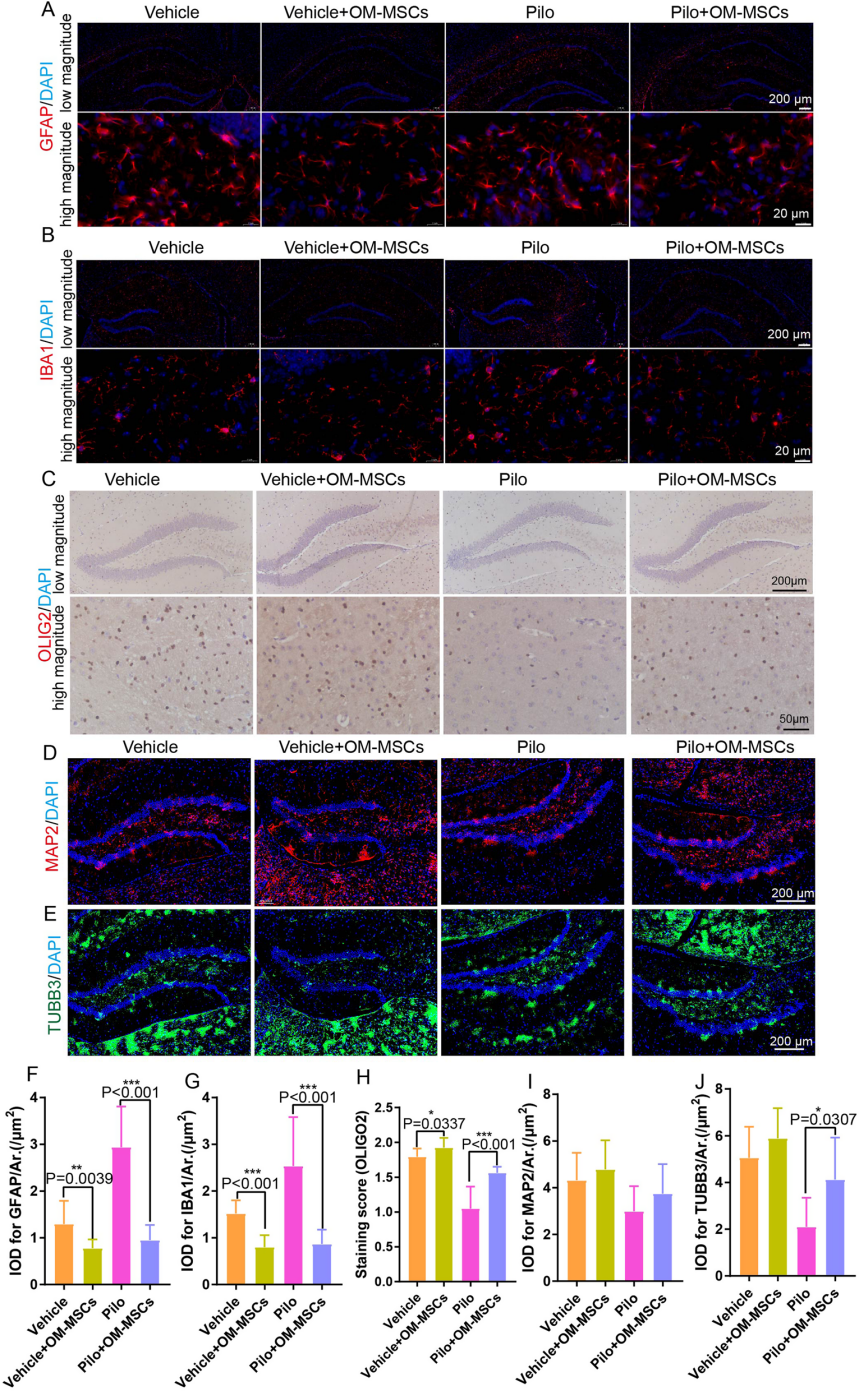
OM-MSC transplant improves the brain microenvironment. **A–C** Representative images of GFAP (red) (**A**), IBA1(red) (**B**) and OLIG2 (brown) (**C**) in hippocampal region of brain tissues from Vehicle, Vehicle + OM-MSCs, Pilo, and Pilo + OM-MSCs group at low magnitude (up) and high magnitude (bottom). **D, E** Representative images of MAP2 (red) (**D**) and TUBB3 (green) (**E**) in hippocampal region of brain tissues from Vehicle, Vehicle + OM-MSCs, Pilo, and Pilo + OM-MSCs group. Nuclei stained with DAPI are shown in blue. **F–J** Quantification of integrated optical density (IOD) for GFAP (**F**), IBA1 (**G**), OLIG2 (**H**) MAP2 (red) (**I**), and TUBB3 (green) (**J**) for panel **A**–**E**

**Fig. 5:**
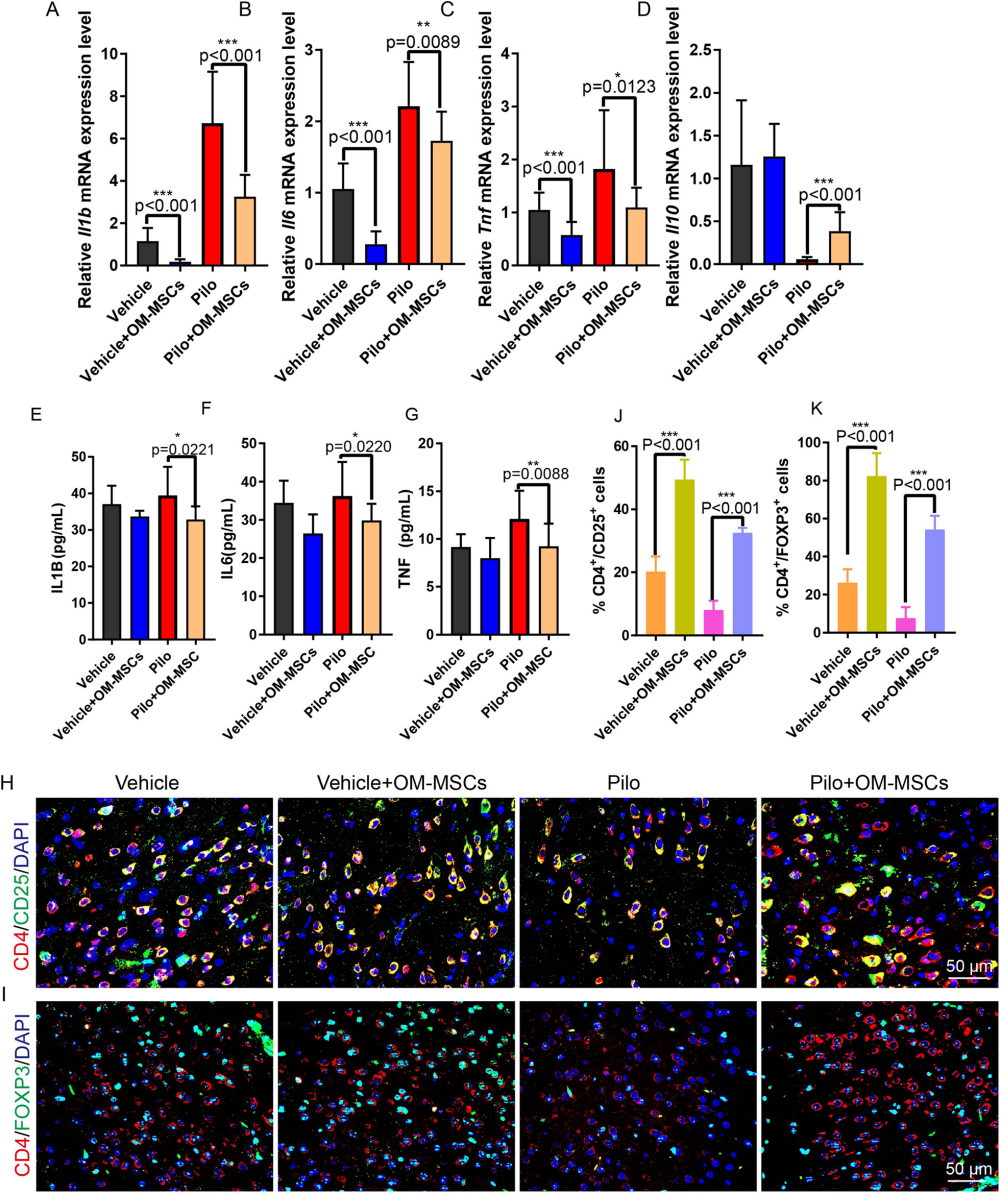
OM-MSCs recruit Treg cells and inhibit the inflammation. **A–D** qRT-PCR evaluates expressions of *Il1b*, *Il6*, *Tnf, Il10* of brain tissue from Vehicle, Vehicle + OM-MSCs, Pilo, and Pilo + OM-MSCs group. *n* = 18 per group. **E–G** ELISA analysis of protein level of IL1B, IL6, and TNF from brain of each indicated group. *n* = 5 per group. **H–K** Representative images and quantification of immunofluorescent staining of double-positive CD4^+^CD25^+^ and CD4^+^FOXP3^+^ cells (Treg cells) from brains of Vehicle, Vehicle + OM-MSCs, Pilo, and Pilo + OM-MSCs group. *n* = 5 per group for CD4^+^CD25^+^ cells and *n* = 11 per group for CD4^+^FOXP3^+^ cells

Brain damage and atrophy are common sequelae after frequent epileptic seizures, and repair of the nervous system is very difficult, which is the major obstacle to cure patients with epilepsy. To determine whether OM-MSC transplantation has beneficial effects on restoring the neuronal cell lose, immunohistochemistry and immunofluorescent staining of each type of nerve cell were performed. Cells with GFAP or IBA1, markers of astrocytes, and microglia, respectively, were lower in the Pilo + OM-MSC group than in the Pilo group, indicating that OM-MSC treatment ameliorated astrogliosis and microgliosis which were attributes of neuroinflammation and injury (Fig. 4A, B, F, G). Cells with OLIG2, MAP2, or TUBB3 markers of oligodendrocytes and neuronal cells, respectively, were higher in the Pilo + OM-MSCs group than in the Pilo group, indicating that OM-MSC treatment ameliorated demyelination and neuronal cell loss conditions (Fig. 4C–E, H–J, Additional file 7: Fig. S7). In addition, we determined the inflammation status of each group. Proinflammatory cytokines such as *Tnf*, *Il1b*, and *Il6* were lower, and the anti-inflammatory cytokine *Il10* was higher in the Pilo + OM-MSC group than in the Pilo group, as determined by both qRT-PCR and ELISA analysis (Fig. 5A–G). These results suggest that the neural network becomes more favorable for brain damage repair.

### OM-MSCs exerted an immunomodulatory effect by recruiting Treg cells

To investigate how OM-MSCs exert an immunomodulatory effect, we found that Treg cells were recruited to the brain, evidenced by a higher number of Treg cells which were evaluated by counting CD4 and FOXP3, CD4, and CD25 double-positive cells in the brain (Fig. 5H–K). This finding is consistent with the previous reports that brain-infiltrating Treg cells are essential for behavioral recovery and brain repair after injury [23]. Our present data suggested that Treg cells were recruited to the injury site of the brain and inhibited inflammation, improving the repairing of neural network.

### OM-MSCs improved the cognitive ability and motor function of an epileptic mouse model

**Fig. 6:**
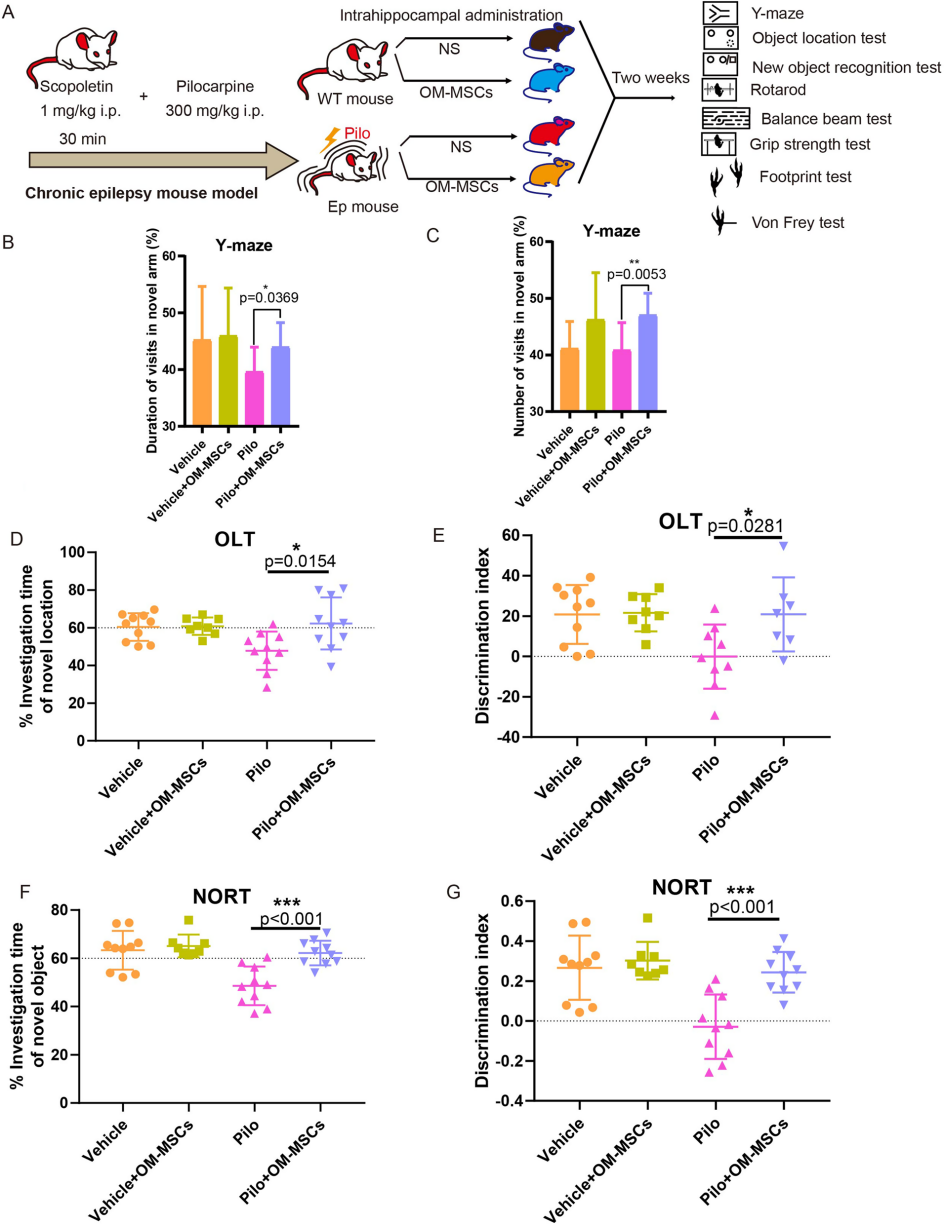
OM-MSC treatment improves cognitive ability in epileptic mice. **A** Schematic representation of experimental procedures. **B–G** Y-maze (**B, C**), LOT (**D, E**), and NOLT (**F, G**) evaluate the cognitive ability of normal, epileptic with or without OM-MSC treatment. *n* = 10 mice per group

**Fig. 7:**
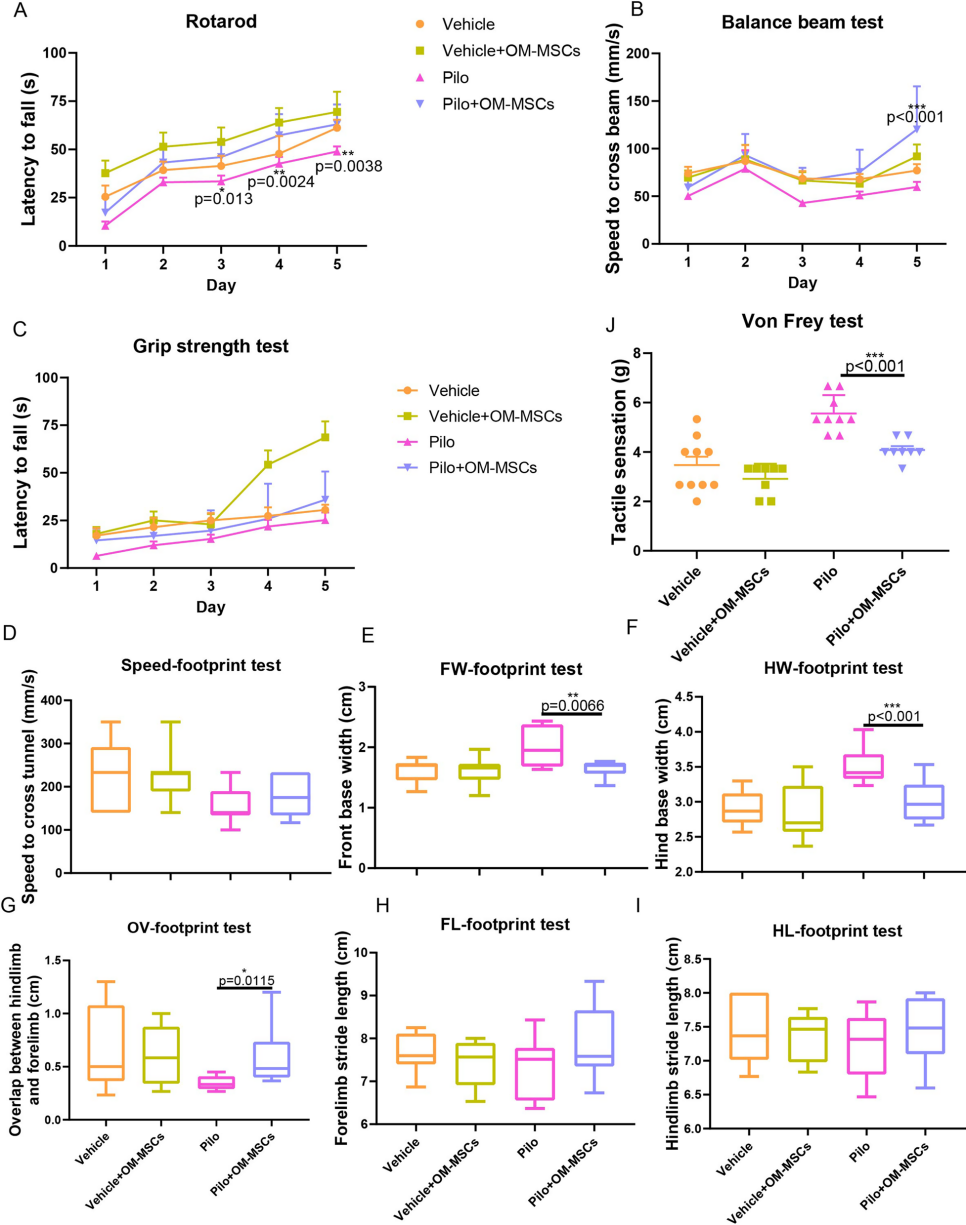
OM-MSC treatment improves motor function and tactile sensation in epileptic mice. **A–C** Movement of mice was evaluated by rotarod test (**A**), balance beam test (**B**), and grip strength test (**C**). **D–I** Movement of mice was evaluated by footprint test. **J** Tactile sensation was evaluated by von Frey test. *n* = 10 per group, three repeats for each mouse. Values were presented as mean ± SD

**Fig. 8:**
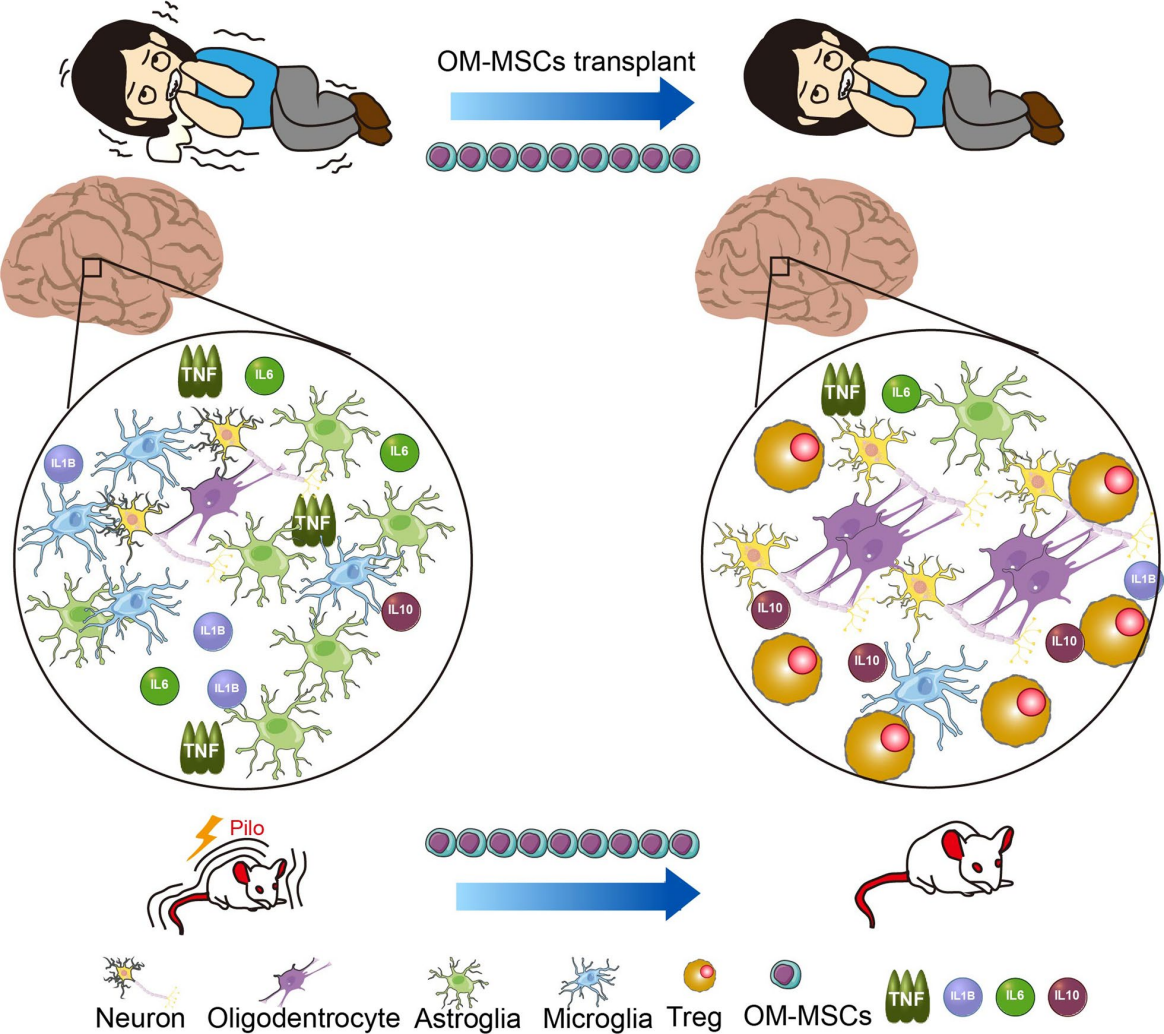
OM-MSC treatment improves cognitive ability in epilepsy. OM-MSCs recruit Tregs, inhibit immune and inflammatory response, rebuild the neural network, and repair brain function

Despite restoring the neuronal cell lose, improving the quality of life is the ultimate goal for the treatment of epilepsy. Behavioral experiments confirmed that OM-MSC treatment improved the memory and cognitive function of an epileptic mouse model (Fig. 6A). Spatial learning, hippocampus-dependent memory, and perirhinal cortex-dependent memory were improved in the Pilo + OM-MSC group compared with the Pilo group, as evidenced by increased duration and the number of visits in the novel arm in the Y-maze test. In the object location and novel object recognition tests, more time is spent investigating the novel location or novel object (Fig. 6B–J). Moreover, improved locomotive function was observed in Pilo mice treated with OM-MSCs. Rotarod and balance beam tests were performed to assess the mice's motor coordination and balance, the grip strength test was conducted to measure muscle tension, and the footprint test was conducted to measure the gaits. As shown in Fig. 7A, the Pilo + OM-MSCs group fell off the rotarod more slowly than the Pilo group. They also had significantly greater grip strength and traversed the balance beam at a much faster speed (Fig. 7B, C). In the footprint test, Pilo + OM-MSC group manifested a narrower base width and longer stride length than the Pilo group (Fig. 7D–I). In addition, the von Frey test was performed to evaluate tactile sensation (Fig. 7J). The Pilo + OM-MSC group revealed a lighter prick threshold than the Pilo group, suggesting improved tactile sensation with OM-MSC treatment. In summary, administration of OM-MSCs indeed caused substantial cognitive, locomotive, and perceptive improvements in Pilo mice (Fig. 8).

## Discussion

Our present study demonstrated that autologous OM-MSC treatment could obviously alleviate brain atrophy and improve seizure symptoms in this chronic refractory epilepsy patient. Additionally, our study reveals that autologous OM-MSC transplantation has beneficial effects on restoring the neuronal cell loss by recruiting Treg cells and inhibiting inflammation.

Previous studies have demonstrated that MSCs are effective in treating epilepsy. Costa-Ferro et al. [24] were the first to indicate the neuroprotective and anti-inflammatory effects of MSCs in epilepsy treatment. However, the quality and efficacy of MSCs vary from different sources. Mesoderm-derived MSCs such as bone marrow mesenchymal stem cells (BM-MSCs), adipose tissue mesenchymal stem cells (AT-MSCs), and umbilical cord mesenchymal stem cells (UC-MSCs) tend to undergo mesodermal differentiation with limited potential for neural differentiation. According to the ClinicalTrials.gov database, three trials (NCT00916266, NCT02497443, NCT03676569) have been completed around the world to study the safety and early efficacy of autologous MSC (BM-MSC or AT-MSC) treatment of epilepsy (Additional file 9: Table S2), suggesting the therapeutic potential of MSC transplantation in epilepsy patients [25–28]. BM-MSCs have been shown to exhibit a rare population (0.001–0.1%) within whole bone marrow cells, slower proliferation rates (40–60 h doubling time), and senescence in early passages (passages 6–7). AT-MSCs are more readily able to exhibit adipogenic differentiation than neural differentiation. Scale-up manufacturing of MSCs provides a great quantity, while shortcomings such as time- and cost-consuming, immunological rejection, and alteration during bioprocessing workflows restrict their application.

OM-MSCs are derived from nasal lamina propria and are considered to be a promising types of MSCs determined in regenerative medicine studies [29]. Compared with other sources of MSCs, OM-MSCs have shown several advantages in recent researches, including simpler protocol for culturing, higher proliferation rate, as well as more favorable for neuronal differentiation and regeneration [10, 11]. Accumulating evidences suggested that OM-MSCs confer neuroprotective effects in various neurological disorders, including cerebral vascular diseases, Parkinson’s disease, and Alzheimer's disease [13, 30–32]. These reported studies indicated that OM-MSCs exerted critical roles in promoting angiogenesis, inhibiting neuronal death and inflammation response as well as improving neurological deficits. More importantly, for MSCs transplanted therapy to humans, the OM-MSCs could be easier received and have lower or no immune rejection due to autologous treatments. Therefore, after approval by the ethics committee and registration of national clinical trials, we decided to conduct her autologous cell transplantation therapy for this patient in the form of clinical treatment case.

A limitation of our study is that we have not addressed whether reversible neural network rebuilding benefits from exogenous OM-MSCs or endogenous stem cells or the transdifferentiation of nerve cells. A study by Munoz et al. [33] showed that human MSCs induce proliferation, migration, and differentiation of the endogenous neural stem cells.

In conclusion, the present data demonstrated that autologous OM-MSC treatment was efficacious for restraining chronic refractory epilepsy, suggesting a future therapeutic candidate for epilepsy.

## Conclusions

OM-MSCs ameliorated the epileptic syndrome by recruiting Treg cells, inhibiting inflammation, and rebuilding the neural network. This is the first experience treating epilepsy with autologous transplantation of OM-MSCs, promoting the remodeling of neural network by regulating immunity (Fig. 8). Clinical and animal experiments have demonstrated that transplantation of OM-MSCs alleviates the symptoms of epilepsy and has great potential and social value for epilepsy treatment.

### Supplementary Information

**Additional file 1**. **Figure S1: **Characterization of human OM-MSCs. Flow cytometric analysis of surface marker gene expressions of human OM-MSCs, characterized as CD44-, CD105-, CD133-, CD146-, CD73-, and CD90-positive and CD45- and CD34-negative.

**Additional file 2**. **Figure S2: **The automatic brain tissue segmentation imaging showing in 2015, 2018, and normal control.** A** Cerebral MRI. **B** White matters segmentation imaging (blue).** C** Gray matters segmentation imaging (red). **D** CSF segmentation imaging (green). Normal control represented a healthy 26-year-old female cerebral MRI.

**Additional file 3**. **Figure S3: **Characterization of mouse OM-MSCs. Flow cytometric analysis of surface marker gene expressions of the isolated OM-MSCs, such as CD73, CD90, CD105, CD34, CD45, and CD31.

**Additional file 4**. **Figure S4: **Tracing the distribution of OM-MSC after stereotaxic delivery by GFP-labeled OM-MSCs**.** Fluorescent images showing the GFP-labeled OM-MSCs in the hippocampus after 7-day and 14-day transplantation.

**Additional file 5**. **Figure S5: **Statistics on amplitude of EEG inFig. S2. n = 5 per group.

**Additional file 6**. **Figure S6: **Postmortem histology of the brain. Comparable sections were chosen, and DAPI staining was performed to show the nuclei in blue. Scale bar: 500 μm.

**Additional file 7**. **Figure S7: **High- and low-magnification images of MAP2 and TUBB3 in hippocampus. Immunofluorescent images showing the MAP2 (red) and TUBB3 (green) in hippocampal region of brain tissues from Vehicle, Vehicle + OM-MSCs, Pilo, and Pilo + OM-MSCs group. Inset (dentate gyrus) in low-magnification images is presented at higher magnification in the bottom panels. Nuclei stained with DAPI are shown in blue. Scale bar: 100 μm (low magnification), 20 μm (high magnification).

**Additional file 8**. **Table S1** Features of the seizures before and after the treatment.

**Additional file 9**. **Table S2** Summary of clinical trials in epilepsy using MSC registered at Clinical Trials. Gov. Search done on February 3, 2022.

## Data Availability

The datasets used and/or analyzed during the current study are available from the corresponding author on reasonable request.

## Abbreviations

OM-MSCs: Olfactory mucosa mesenchymal stem cells
MRI: Magnetic resonance imaging
AEDs: Anti-epileptic drugs
GTCSs: Generalized tonic–clonic seizures
AEEG: Ambulatory electroencephalography
RE: Refractory epilepsy
AS: Absence seizure
TCV: Total cerebral volume
CSF: Cerebrospinal fluid
OLT: Object location test
NORT: Novel object recognition test
BM-MSCs: Bone marrow mesenchymal stem cells
AT-MSCs: Adipose tissue mesenchymal stem cells
UC-MSCs: Umbilical cord mesenchymal stem cells
