# Supplementary figures and images for: Autologous olfactory mucosa mesenchymal stem cells treatment improves the neural network in chronic refractory epilepsy
Source: Stem Cell Res Ther. 2023 Sep 7;14:237. doi: 10.1186/s13287-023-03458-6 (PMC10483711; doi:10.1186/s13287-023-03458-6)

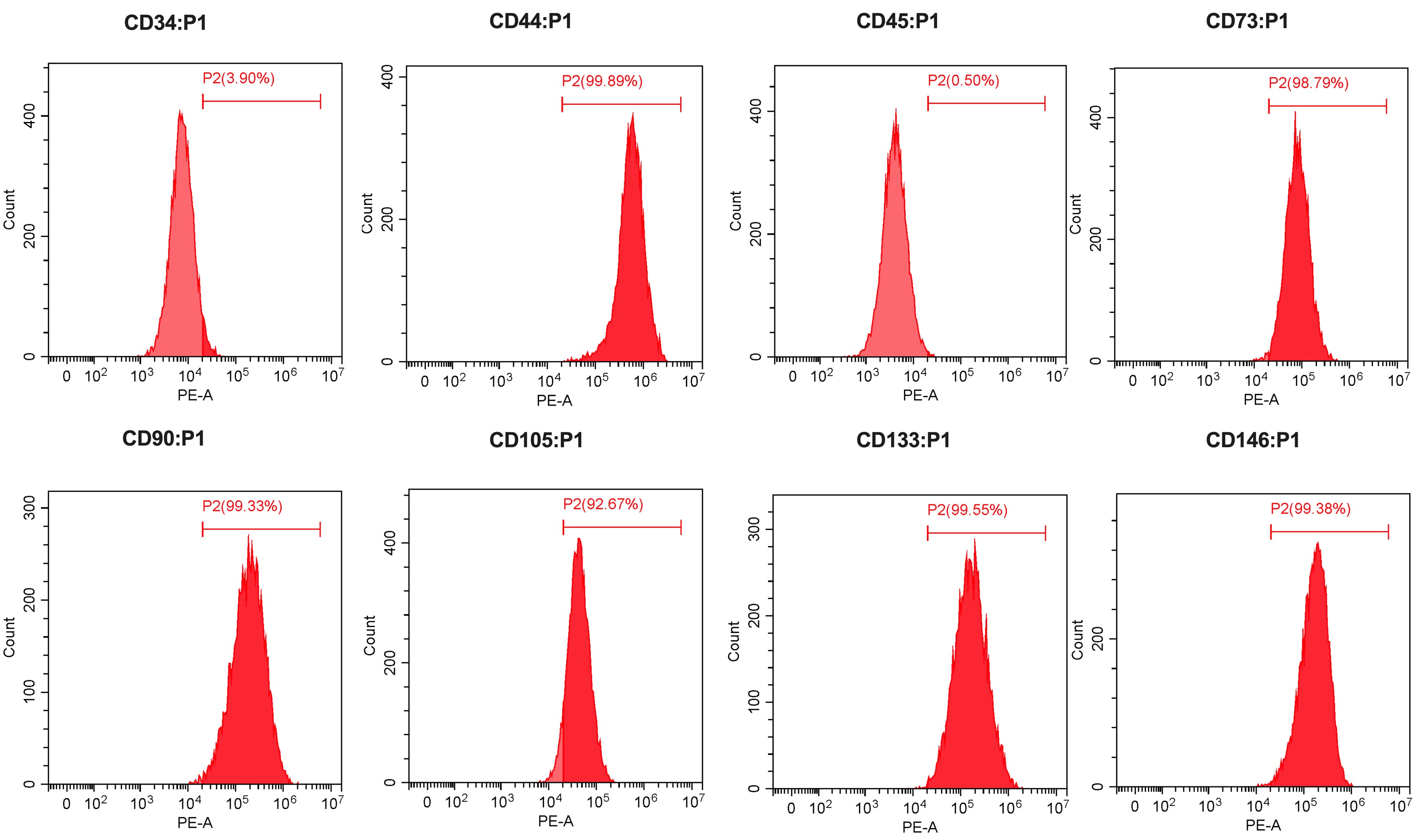

Supplement: Supplementary file 1 — Additional file 1. Figure S1: Characterization of human OM-MSCs. Flow cytometric analysis of surface marker gene expressions of human OM-MSCs, characterized as CD44-, CD105-, CD133-, CD146-, CD73-, and CD90-positive and CD45- and CD34-negative. [file 13287_2023_3458_MOESM1_ESM.jpg]

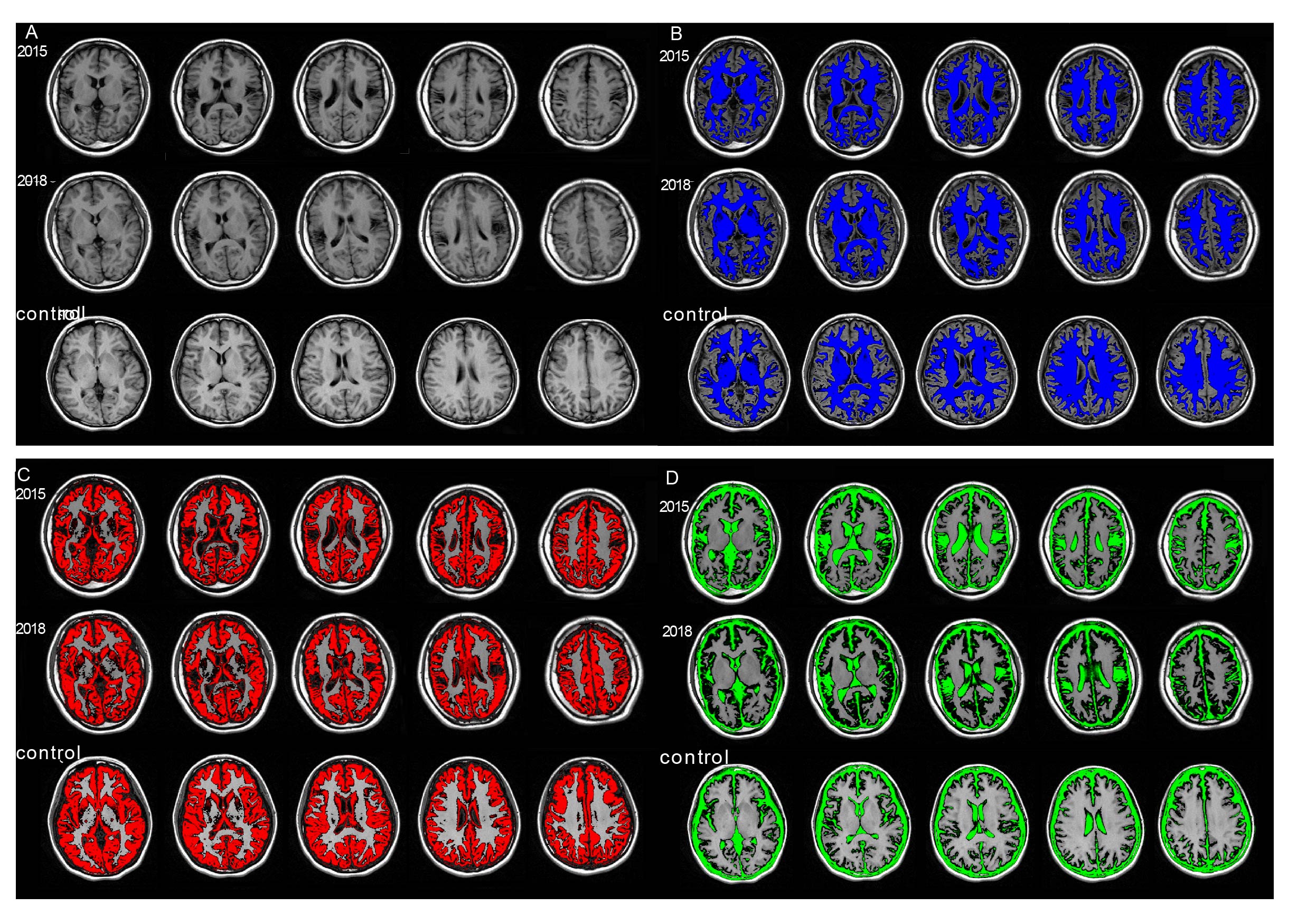

Supplement: Supplementary file 2 — Additional file 2. Figure S2: The automatic brain tissue segmentation imaging showing in 2015, 2018, and normal control. A Cerebral MRI. B White matters segmentation imaging (blue). C Gray matters segmentation imaging (red). D CSF segmentation imaging (green). Normal control represented a healthy 26-year-old female cerebral MRI. [file 13287_2023_3458_MOESM2_ESM.jpg]

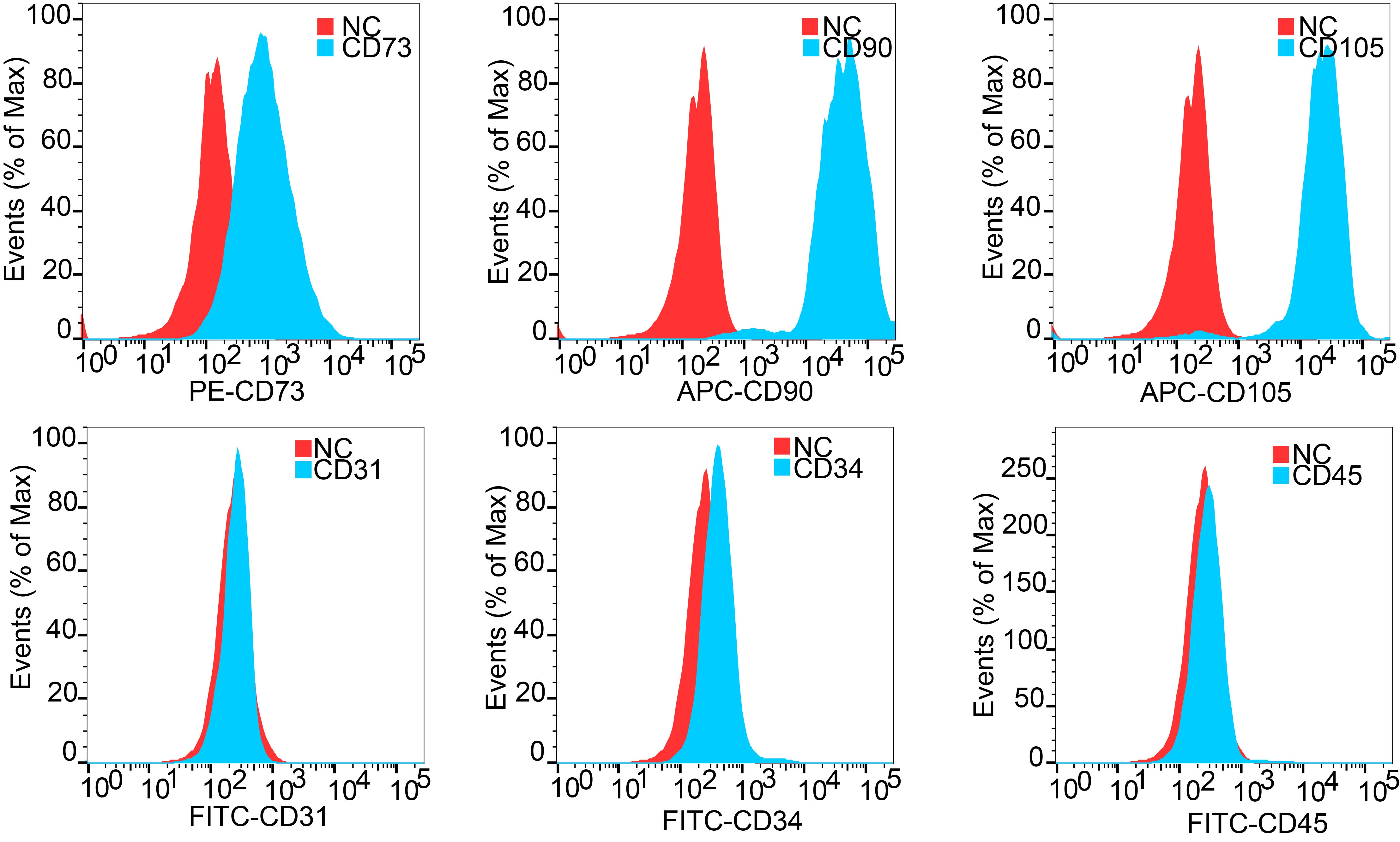

Supplement: Supplementary file 3 — Additional file 3. Figure S3: Characterization of mouse OM-MSCs. Flow cytometric analysis of surface marker gene expressions of the isolated OM-MSCs, such as CD73, CD90, CD105, CD34, CD45, and CD31. [file 13287_2023_3458_MOESM3_ESM.jpg]

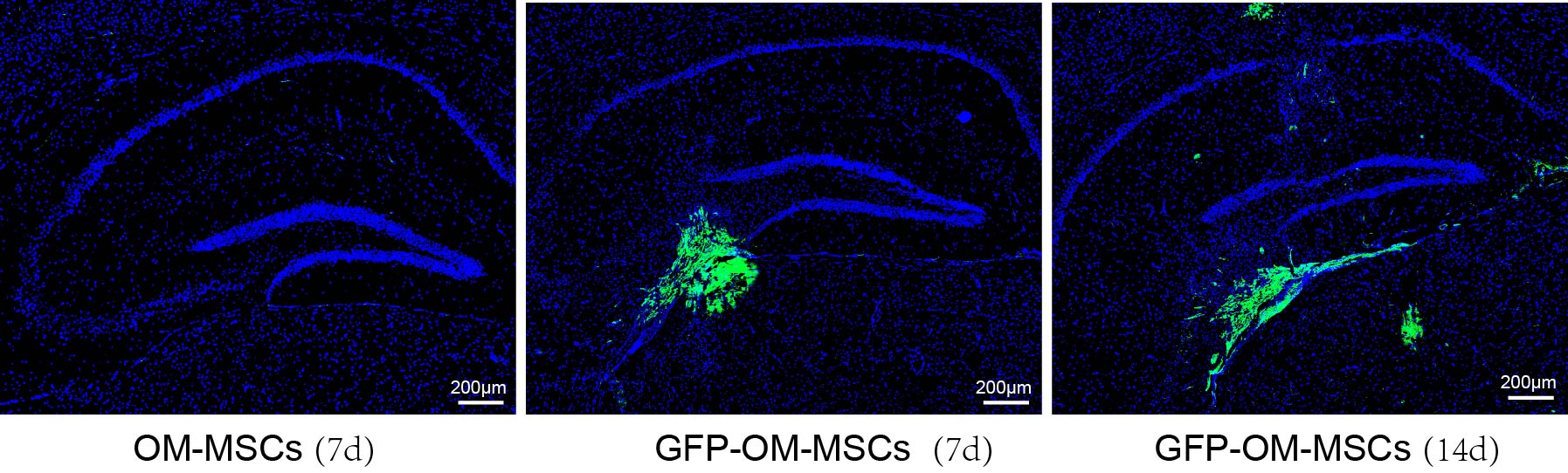

Supplement: Supplementary file 4 — Additional file 4. Figure S4: Tracing the distribution of OM-MSC after stereotaxic delivery by GFP-labeled OM-MSCs. Fluorescent images showing the GFP-labeled OM-MSCs in the hippocampus after 7-day and 14-day transplantation. [file 13287_2023_3458_MOESM4_ESM.jpg]

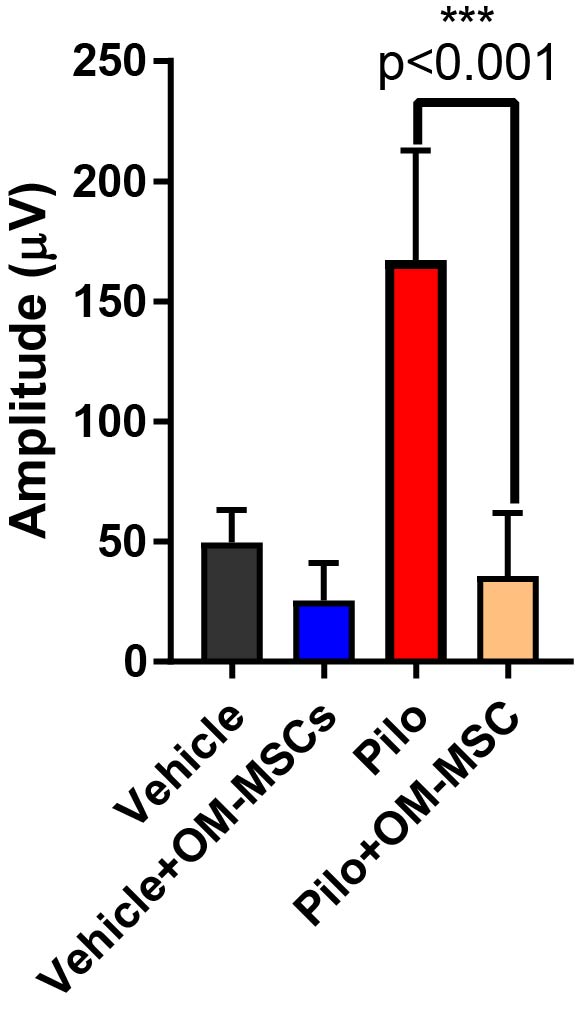

Supplement: Supplementary file 5 — Additional file 5. Figure S5: Statistics on amplitude of EEG inFig. S2. n = 5 per group. [file 13287_2023_3458_MOESM5_ESM.jpg]

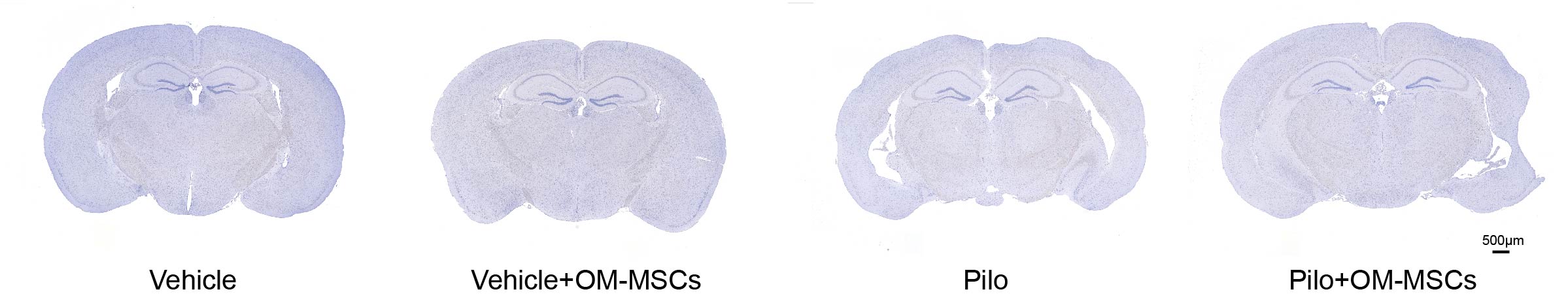

Supplement: Supplementary file 6 — Additional file 6. Figure S6: Postmortem histology of the brain. Comparable sections were chosen, and DAPI staining was performed to show the nuclei in blue. Scale bar: 500 μm. [file 13287_2023_3458_MOESM6_ESM.jpg]

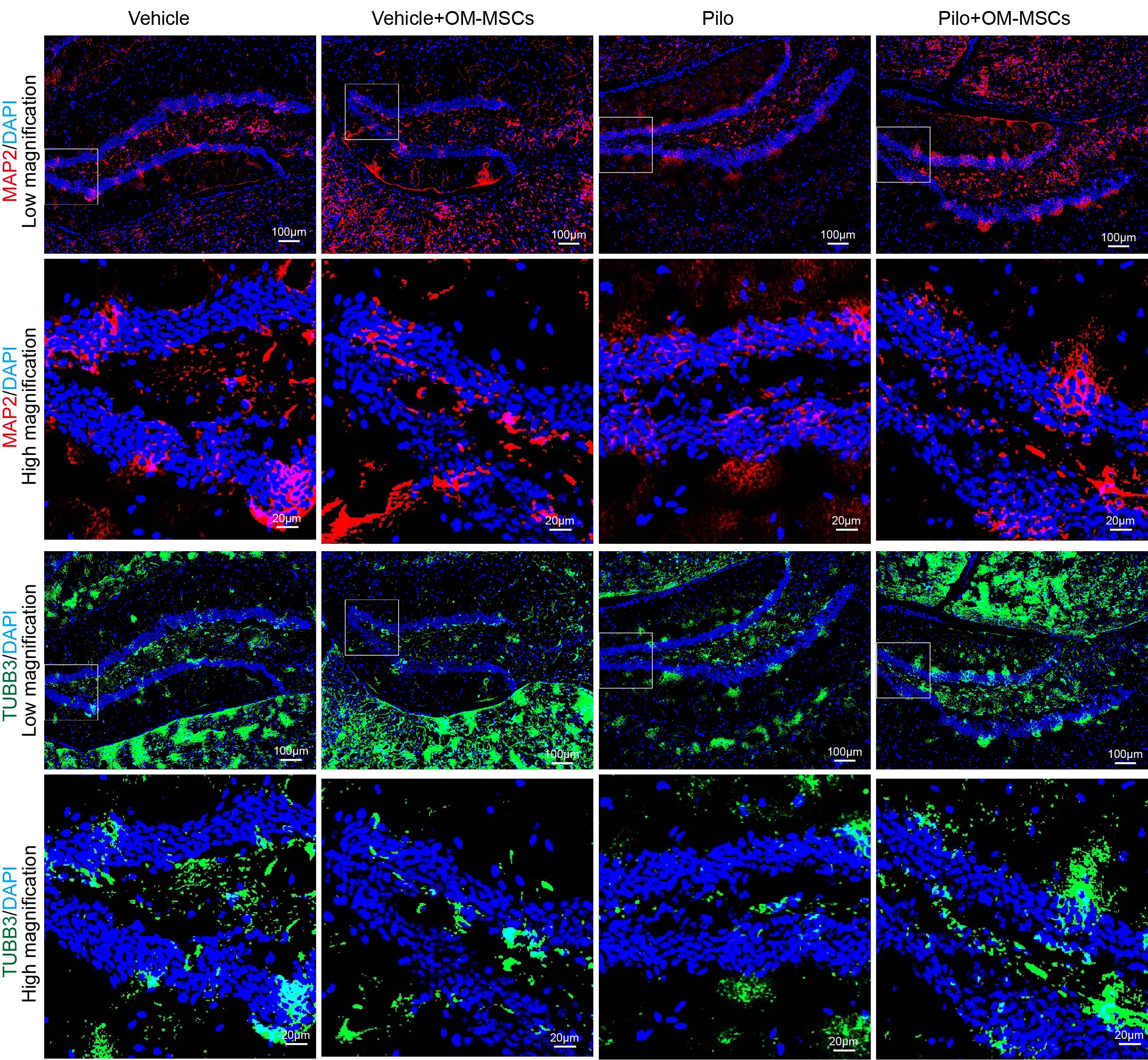

Supplement: Supplementary file 7 — Additional file 7. Figure S7: High- and low-magnification images of MAP2 and TUBB3 in hippocampus. Immunofluorescent images showing the MAP2 (red) and TUBB3 (green) in hippocampal region of brain tissues from Vehicle, Vehicle + OM-MSCs, Pilo, and Pilo + OM-MSCs group. Inset (dentate gyrus) in low-magnification images is presented at higher magnification in the bottom panels. Nuclei stained with DAPI are shown in blue. Scale bar: 100 μm (low magnification), 20 μm (high magnification). [file 13287_2023_3458_MOESM7_ESM.jpg]

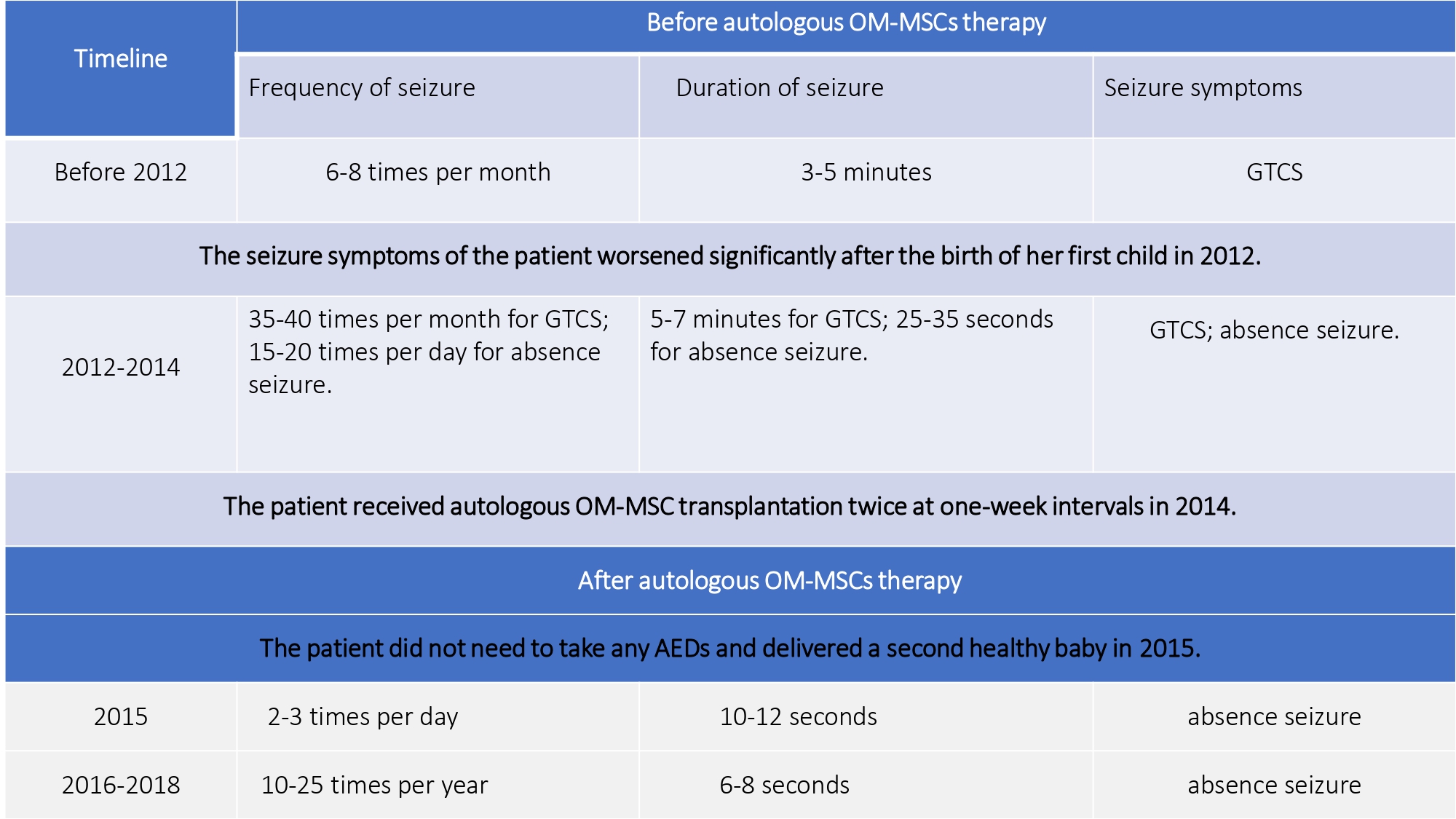

Supplement: Supplementary file 8 — Additional file 8. Table S1 Features of the seizures before and after the treatment. [file 13287_2023_3458_MOESM8_ESM.jpg]

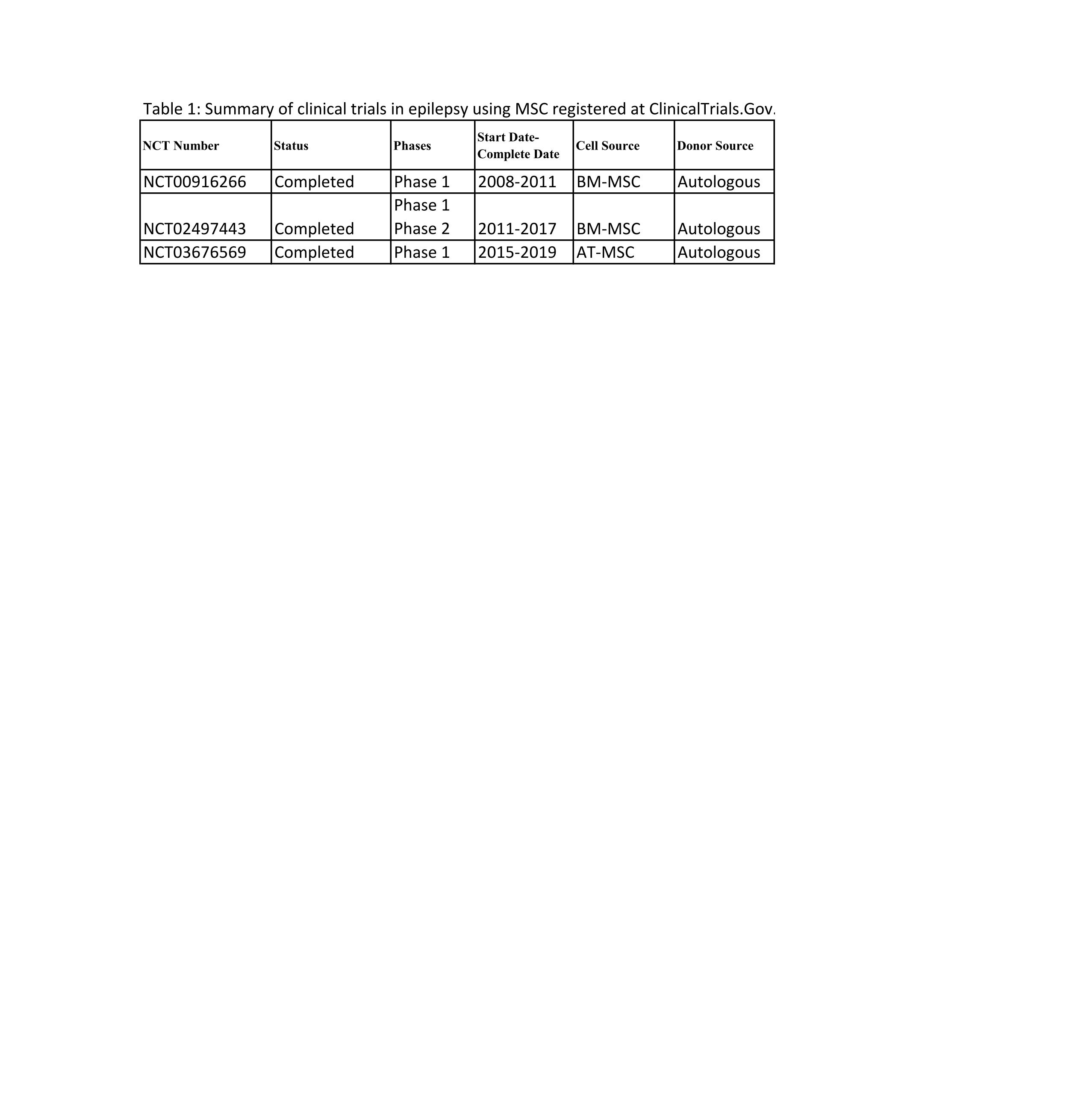

Supplement: Supplementary file 9 — Additional file 9. Table S2 Summary of clinical trials in epilepsy using MSC registered at Clinical Trials. Gov. Search done on February 3, 2022. [file 13287_2023_3458_MOESM9_ESM.jpg]
